# Landscape of pharmacogenetic variants associated with non-insulin antidiabetic drugs in the Indian population

**DOI:** 10.1136/bmjdrc-2023-003769

**Published:** 2024-03-12

**Authors:** Ambily Sivadas, S Sahana, Bani Jolly, Rahul C Bhoyar, Abhinav Jain, Disha Sharma, Mohamed Imran, Vigneshwar Senthivel, Mohit Kumar Divakar, Anushree Mishra, Arpita Mukhopadhyay, Greg Gibson, KM Venkat Narayan, Sridhar Sivasubbu, Vinod Scaria, Anura V Kurpad

**Affiliations:** 1 St John's Research Institute, Bangalore, Karnataka, India; 2 CSIR Institute of Genomics and Integrative Biology, New Delhi, India; 3 Academy of Scientific and Innovative Research (AcSIR), Ghaziabad, Uttar Pradesh, India; 4 Georgia Institute of Technology, Atlanta, Georgia, USA; 5 Rollins School of Public Health, Atlanta, Georgia, USA; 6 St John's Medical College, Bangalore, Karnataka, India

**Keywords:** Indians, Diabetes Mellitus, Type 2, Genomics

## Abstract

**Introduction:**

Genetic variants contribute to differential responses to non-insulin antidiabetic drugs (NIADs), and consequently to variable plasma glucose control. Optimal control of plasma glucose is paramount to minimizing type 2 diabetes-related long-term complications. India’s distinct genetic architecture and its exploding burden of type 2 diabetes warrants a population-specific survey of NIAD-associated pharmacogenetic (PGx) variants. The recent availability of large-scale whole genomes from the Indian population provides a unique opportunity to generate a population-specific map of NIAD-associated PGx variants.

**Research design and methods:**

We mined 1029 Indian whole genomes for PGx variants, drug–drug interaction (DDI) and drug–drug–gene interactions (DDGI) associated with 44 NIADs. Population-wise allele frequencies were estimated and compared using Fisher’s exact test.

**Results:**

Overall, we found 76 known and 52 predicted deleterious common PGx variants associated with response to type 2 diabetes therapy among Indians. We report remarkable interethnic differences in the relative cumulative counts of decreased and increased response-associated alleles across NIAD classes. Indians and South Asians showed a significant excess of decreased metformin response-associated alleles compared with other global populations. Network analysis of shared PGx genes predicts high DDI risk during coadministration of NIADs with other metabolic disease drugs. We also predict an increased CYP2C19-mediated DDGI risk for CYP3A4/3A5-metabolized NIADs, saxagliptin, linagliptin and glyburide when coadministered with proton-pump inhibitors (PPIs).

**Conclusions:**

Indians and South Asians have a distinct PGx profile for antidiabetes drugs, marked by an excess of poor treatment response-associated alleles for various NIAD classes. This suggests the possibility of a population-specific reduced drug response in atleast some NIADs. In addition, our findings provide an actionable resource for accelerating future diabetes PGx studies in Indians and South Asians and reconsidering NIAD dosing guidelines to ensure maximum efficacy and safety in the population.

**WHAT IS ALREADY KNOWN ON THIS TOPIC**

- Response to antidiabetes therapy is known to be influenced by drug–gene (pharmacogenetic (PGx)), drug-drug and drug–drug–gene interactions.
- Most diabetes patients (60%–78%) experience poor glycemic control in India; however, there is a dearth of comprehensive studies assessing the prevalence of such interactions in the Indian population.

**WHAT THIS STUDY ADDS**

- We identified 76 known and 52 common predicted deleterious PGx variants associated with 44 non-insulin antidiabetic drugs in Indians, showing significant allele frequency differences with other global populations.
- We observed a significant excess of decreased metformin response-associated alleles in Indians and South Asians compared with other global populations suggesting likely decreased metformin response.

**HOW THIS STUDY MIGHT AFFECT RESEARCH, PRACTICE OR POLICY**

- Our findings offer valuable insights including a comprehensive resource of known and predicted pharmacogenomic markers relevant to prioritizing future diabetes PGx studies in Indians and South Asians.

## Introduction

Type 2 diabetes is epidemic in India, affecting over 101 million patients^1^ and recording the highest increase in mortality rate among non-communicable diseases during 1990–2016.^2^ This is substantially attributed to poor glycemic control in the long term, leading to microvascular and macrovascular complications. Non-insulin antidiabetic drugs (NIADs) remain the mainstay of glycemic control in type 2 diabetes therapy. Several classes of NIADs are available including biguanides (metformin), sulfonylureas (SU), thiazolidinediones (TZDs), α-glucosidase inhibitors, dipeptidyl peptidase 4 (DPP-4) inhibitors, glucagon-like peptide-1 (GLP-1) analogs, sodium-glucose co-transporter 2 (SGLT2) inhibitors and meglitinides. However, there is remarkable interindividual variation in hypoglycemic response.^3^ Reported monotherapy failures at 5 years also vary significantly between different drug classes; being highest with glyburide (34%), followed by metformin (21%) and rosiglitazone (15%).^4^ Most Indian patients (60%–78%) experience poor glycemic control,^5 6^ aligned with reports from across the world,^7^ and this is linked to 5-year mortality in new-onset type 2 diabetes patients.^8^ An important target of effective therapy is precision based on individual pharmacokinetics/dynamics (PK-PD).

Type 2 diabetes is a chronic metabolic disease influenced by genetic and environmental factors and characterized by impaired insulin secretion and insulin resistance. Pharmacogenomic studies have identified hundreds of genetic variants that influence individual PK-PD profiles, causing varied glycemic control with several NIADs, including metformin.^9 10^ However, only a few of these reported variants have been validated in the Indian population.^11–13^ In addition, the small effect sizes of these variants underline the limitations of clinically translating a single variant’s impact on treatment response in contrast to emerging polygenic score-based approaches.

Diabetes patients experience poor treatment outcomes also due to drug–drug interactions (DDIs) associated with polypharmacy. The magnitude of DDIs varies considerably among patients owing to genetic variations, leading to drug–drug–gene interactions (DDGIs). One emblematic example of genotype-dependence of DDGIs is that of > threefold difference in increase in tacrolimus (CYP2C19/3A4 substrate) exposure when coadministered with voriconazole (CYP2C19/3A4 inhibitor and substrate) in a CYP2C19 poor metabolizer (1500%) versus CYP2C19 intermediate metabolizer (400%).^14^ Clinical studies suggest that DDIs, drug–gene interactions (PGx) and DDGIs contribute to 53%–66%, 15%–25% and 19%–22% of all drug interactions.^15 16^ Therefore, precision therapy for NIADs would be incomplete without considering the impact of PGx polymorphisms on drug interactions.

India’s first large-scale whole genome sequencing project (CSIR-IndiGen) has compiled extensive genetic variation data across 1029 individuals belonging to diverse ethnic groups of India.^17^ We recently used this data set to define a comprehensive PGx landscape of Indians across drug categories^18^ and specifically for COVID-19 therapy.^19^ However, no such systematic investigation has been carried out for NIADs, especially with a focus on the cumulative burden of associated PGx variants. This study aimed to create a population-specific map of NIAD-associated PGx variants in Indians using whole genomes. The differential cumulative occurrence of known PGx variants among Indians was compared with other populations. We also explored DDIs and DDGIs associated with polypharmacy in diabetes care.

## Methods

### Drugs used in diabetes therapy

A comprehensive list of 44 NIADs across eight drug classes such as ⍺-glucosidase inhibitors, biguanides, DPP-4 inhibitors, GLP-1 analogs, SGLT2 inhibitors, sulfonylureas, TZDs and meglitinides was obtained from Drugbank database (online supplemental table S1).

10.1136/bmjdrc-2023-003769.supp1 Supplementary data

### Study population and datasets

Genetic variation data belonging to 1029 unrelated Indians were obtained from whole genomes sequenced in IndiGen^17^ that consisted of 55 898 112 variations mapped to GRCh38 reference genome. A final set of 53 672 515 variations was obtained after quality control. Star alleles were derived for different pharmacogenes (online supplemental methods). The Indian variant allele frequencies (AFs) were obtained from IndiGen project.^20^ The haplotype AFs were estimated using in-house scripts (https://github.com/Sahana-selvarathnam/AF-calculation/).

### Pharmacogenomic variant analysis workflow

We used ANNOVAR^21^ to annotate variants using RefGene gene annotations and dbSNP (V.150) variation database. Variant AFs across global populations in 1000 Genomes Phase 3 (1KGP3),^22^ gnomAD^23^ and Greater Middle East (GME) variome^24^ databases were also compiled using the tool. The exonic variants were assessed for their functional impact to identify potentially deleterious variants (online supplemental methods). Known NIAD-associated PGx variants were obtained using PharmGKB and population-wise AFs were compared (online supplemental methods).

### Cumulative allele count analysis

The known PGx clinical annotations were classified as positive or negative impact if the variant minor allele in Indians leads to an improved or reduced treatment outcome, respectively, as per the PharmGKB clinical annotation. For each drug class, the positive and negative allele counts were aggregated separately in each individual using the genotype data from IndiGen and 1000 Genomes projects after LD pruning (r^2^ threshold of 0.2) using SNPClip.^25^ In case of a genetic variant associating with the efficacy as well as toxicity of a drug (ie, decreased response/decreased toxicity or increased response/increased toxicity), the direction of impact on the drug efficacy is considered for the classification. The allele counts were then plotted and compared across populations using the ggboxplot function in the ggpubr R package.^26^ The mean allele counts across all the population groups were compared using Kruskal-Wallis test while the mean allele counts in IndiGen were compared with each of the 1KGP3 super populations in a pairwise manner using the Wilcoxon test.

### Annotation of predicted PGx variants

All pharmacogenes associated with the selected NIADs were obtained from Drugbank and overlapped with predicted deleterious variants in IndiGen to compile potentially deleterious NIAD-associated PGx variants in Indians. AFs of the most common predicted deleterious PGx variants (AF >1%) in Indians were compared across populations. Flourish studio tool^27^ was used to visualize a sankey plot of the drug function disruption pathway.

### DDI and DDGI analysis

To identify DDIs during polypharmacy in diabetes patients, a comprehensive list of 242 drugs indicated for various metabolic disorders were compiled from Drugbank. The pharmacogenes associated with metabolic drug classes and NIADs were overlapped and visualized using UpSetR package.^28^ Drug–gene associations for NIADs and metabolic disease drugs were depicted as a Cytoscape network with gene and drug label sizes proportional to their node degree and drug interaction scores, respectively (online supplemental methods). The CYP inhibitor data were obtained by overlapping 44 NIADs and 242 metabolic disease drugs with the Flockhart table of drug–gene interactions. Known DDGIs were primarily compiled from recent review studies.^14 29 30^

## Results

### Antidiabetic drug list and genetic variation datasets

A master set of 44 NIADs belonging to eight different drug classes such as ⍺-glucosidase inhibitors, biguanides, DPP-4 inhibitors, GLP-1 analogs, SGLT2 inhibitors, sulfonylureas, TZDs and meglitinides was curated from PharmGKB^31^ and Drugbank^32^ databases (online supplemental table S1). Ninety-four genes were found to be associated with their PK/PD through the function of an enzyme, transporter and/or target in Drugbank.

The primary Indian genetic variation data set consists of 1029 whole genomes obtained from unrelated resident Indian individuals belonging to IndiGen (hereafter referred to as Indians).^17^ This data set has catalogued 53 672 515 variants that include single nucleotide variations (SNVs) and insertion/deletions. AFs were estimated from IndiGen and other global population variation data sets such as 1KGP3(22), gnomAD^23^ and GME.^24^ The non-resident Indian, Bangladeshi, Pakistani and Sri Lankan populations (N=489) represented by SAS superpopulation in 1KGP3 will hereafter be referred to as South Asians.

### Known PGx variants associated with NIADs in Indians

**Figure 1:**
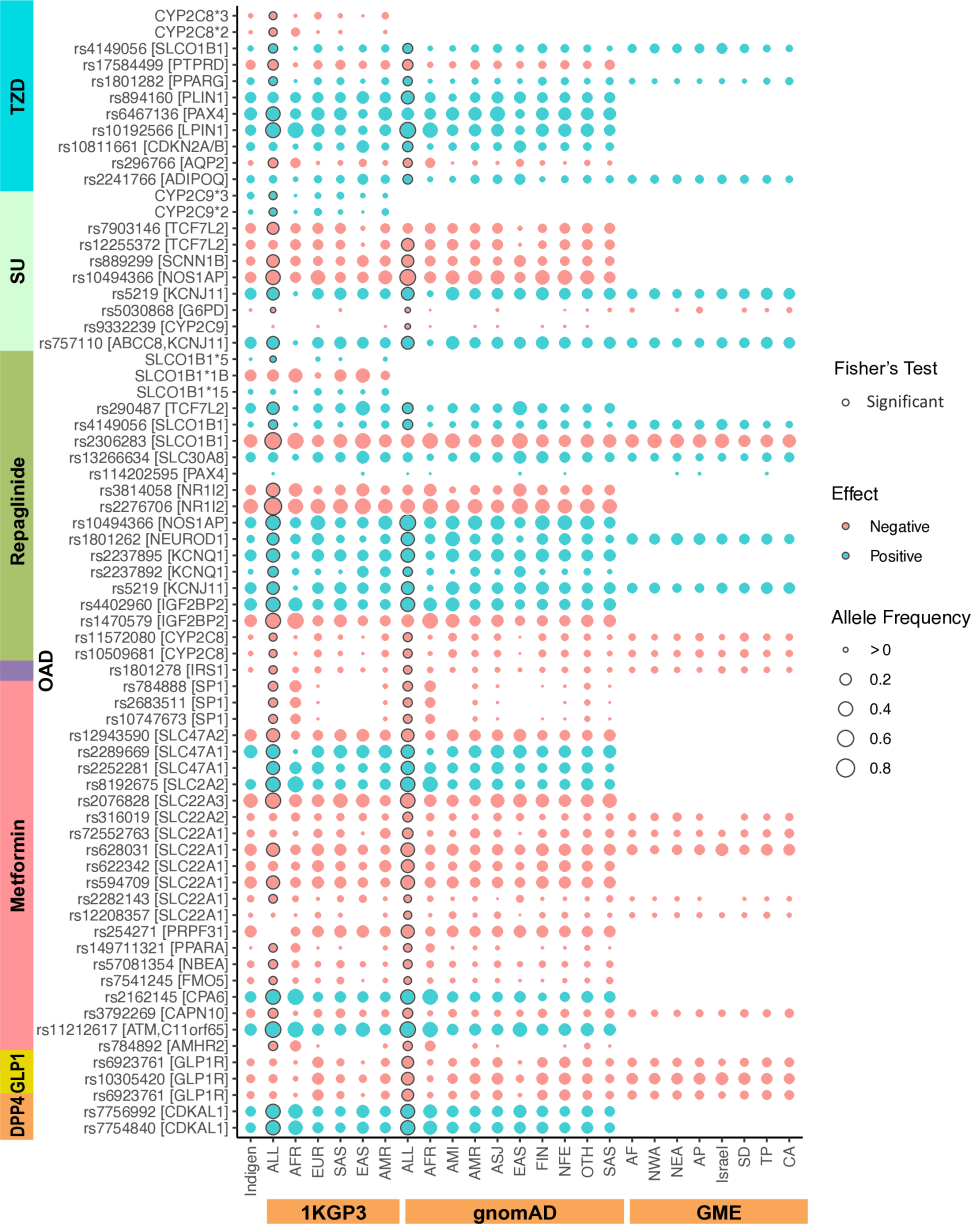
Distribution of NIAD-associated PharmGKB variants in Indians and other global populations. A bubble plot comparing Indian allele frequencies of known NIAD-associated PGx variants with populations in 1000 Genomes project, gnomAD database and GME database. The variant bubbles are colored according to their direction of impact (red: negative/decreased response-associated variants and green: positive/increased response-associated variants). PGx variants showing significant allele frequency differences (Fisher’s exact test, fdr-corrected, p<0.05) between Indians and the global average represented by 1KGP3-ALL and gnomAD-ALL are highlighted with a black outer circle. OAD, Oral antidiabetes drugs; GME, Greater Middle East; NIAD, non-insulin antidiabetic drug; PGx, pharmacogenetic.

A set of 75 drug-variant clinical annotations from PharmGKB overlapped with our master NIAD list involving 58 SNVs and 7 haplotype variants. We observed that no clinically significant SNV annotations (high (level 1) or moderate (level 2) evidence^33^ have been reported for any of the NIADs. Seventy-two of them have level 3 evidence while the remaining three have level 4 evidence which are not strong enough to be used for clinical translation yet owing to those studies having small sample sizes and/or not been replicated. Biguanides (metformin) showed the highest number of PGx associations (N=24) followed by sulfonylureas (N=15), glinides (repaglinide, N=17) and TZDs (N=13). The variant AFs showed remarkable differences between Indians and other global populations (figure 1, online supplemental table S2). For example, *CPA6* variant rs2162145 that is associated with better metformin response showed over threefold variation in its prevalence (AF) across populations ranging from 25% to 34% in Europeans and Indians, respectively, to 82% among Africans. Similarly, reduced pioglitazone efficacy-associated *PTPRD* variant rs17584499 varies from 3% AF among Africans to 24% among Indians. All variants excluding two (rs114202595 and rs13266634) showed significant frequency differences (p<0.05, Fisher’s test, fdr-corrected) between Indians and other global populations (gnomAD-ALL/1KGP3-ALL).

10.1136/bmjdrc-2023-003769.supp2 Supplementary data

Genetic variants associated with decreased metformin response such as *SLC22A1* variants, rs622342 (C), rs628031 (A) and rs594709 (G) and *SLC47A2* variant rs12943590 (A) showed AFs of 25%, 40%, 40% and 40%, respectively, in Indians. The frequencies of rs11212617 (C) in *ATM* gene, rs8192675 variant (C) in *SLC2A2* gene and rs2289669 variant in *SLC47A1* gene (A) associated with better metformin response were 41%, 28% and 51%, respectively, among Indians. Only 26% of Indians carried at least one copy of better sulfonylurea response associated CYP2C9*2 and CYP2C9*3 alleles while 62% of Indians carried GG genotype at *TCF7L2* variant rs12255372 that favourably influences SU treatment success (OR=1.95^34^). Similarly, another better SU response-associated variant rs5219 (T) in *KCNJ11* gene showed a prevalence of 40% among Indians. In case of TZDs, *PPARG* variant rs1801282 (G), which is associated with better response to pioglitazone, was found at 12% AF among Indians compared with a global average of 7% (1KGP3-ALL).

Additionally, 12% of Indians carry at least one copy of *GLP1R* variant rs6923761(A) that is associated with decreased response to DPP-4I (sitagliptin or vildagliptin) and GLP1RA (liraglutide) treatment. 17.6% of Indians also harbor the decreased liraglutide response-associated *GLP1R* polymorphism, rs10305420(T). Interestingly, *KCNQ1* variant rs2237892, which is associated with improved response to repaglinide in Chinese patients, was found at significantly lower frequency among Indians (4%) compared with the global average (1KGP3-ALL: 15%, gnomAD-ALL: 9%). Furthermore, the prevalence of CYP2C8*3 allele, that is associated with increased metabolism of repaglinide, was found to be 3.3% among Indians compared with 12% in Europeans. Finally, *IRS1* variant rs1801278, which is associated with an overall poor response to NIADs, was observed in 3% of Indians compared with a global average of 5%. Interestingly, the highest AF for this variant was observed among Israelis in GME database (10%).

**Figure 2:**
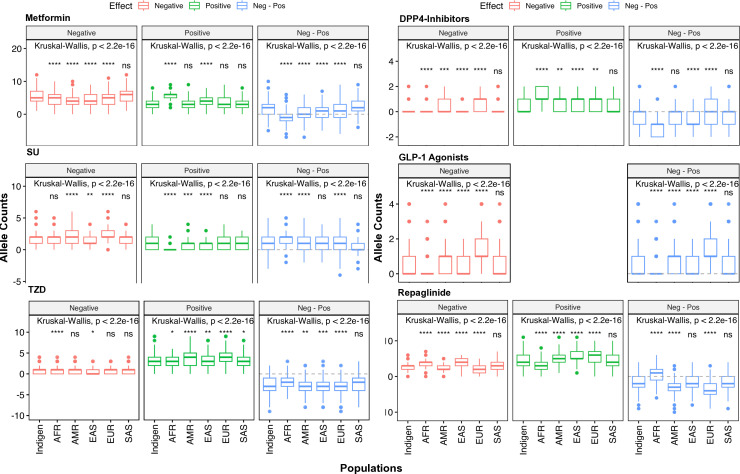
Population-wise cumulative allelic burden of NIAD-associated PGx SNPs. A box plot representation of the cumulative allele counts of known PGx variants associated with different NIAD classes in Indians and other 1KGP3 super populations sorted by their direction of impact. The difference between the negative and positive allele counts is also plotted in the third panel for each NIAD category. The mean allele counts from IndiGen were compared pairwise with the remaining super populations using the Wilcoxon test and significant differences are indicated. Kruskal-Wallis test was used to perform an overall comparison of the mean allele counts across multiple population groups. AFR, African; AMR, Admixed American; EAS, East Asian; EUR, European; NIAD, non-insulin antidiabetic drug; SNP, Single Nucleotide Polymorphism.

We also assessed the differential prevalence of PGx variants categorized and aggregated by the direction of variant impact (negative or positive) on drug response (figure 2). The clinical annotations indicated that variants are associated with 31 improved therapeutic outcomes (positive) and 42 reduced response outcomes (negative) to various NIADs. The combinatorial occurrence (allele counts) of positive and negative-impact alleles per individual sorted by different NIAD classes were then compared between populations. The median count of metformin-associated (positive/negative) alleles per individual was found to be 7 in Admixed Americans, 9 in Indians and South Asians and 10 in Africans. For SUs, the total response-associated allele counts per individual varied from two in Africans and East Asians to three in others. All populations showed an average total count of 4 TZD-associated allele counts per individual. A total of nine repaglinide-associated alleles per individual were observed in East Asians while Indians, South Asians and African Americans harbored a median count of seven alleles.

Overall, Indians showed significant disparities in the mean negative and positive cumulative allele counts compared with every 1KGP3 superpopulation except South Asians across NIAD classes. In case of metformin, negative allele counts were remarkably higher than positive allele counts among Indians in contrast to Africans who had more positive allele counts over negative alleles suggesting substantial altered metformin response in Indians. The median positive-impact cumulative allele counts associated with repaglinide response outnumbered negative allele counts by four in Europeans compared with two in Indians and South Asians while Africans showed a contrasting trend. A careful inspection of this differential variant burden based on the direction of impact further underscores the importance of assessing the combinatorial polygenic effect.

### Potentially deleterious NIAD-associated PGx variants in Indians

**Figure 3:**
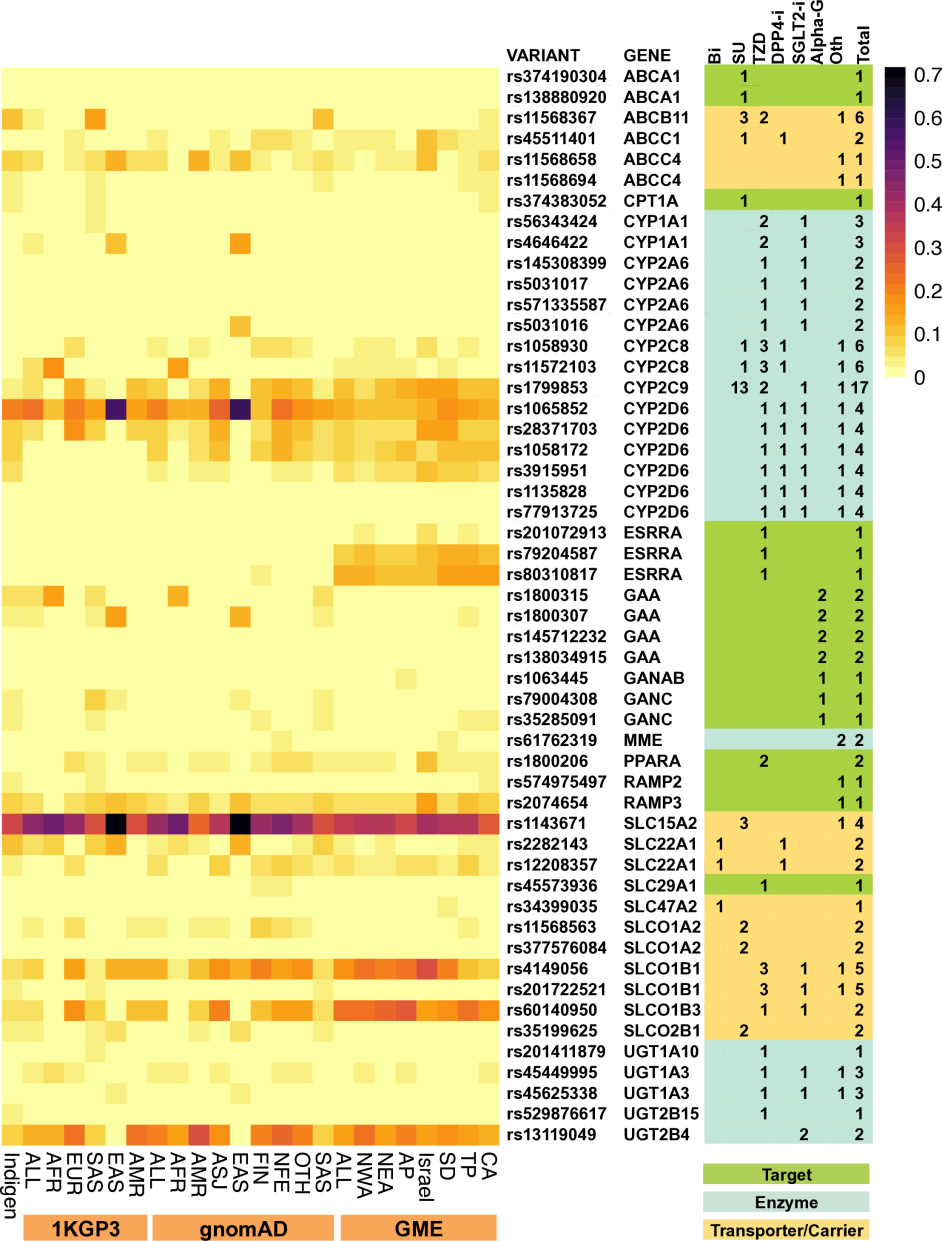
Distribution of common predicted deleterious NIAD-associated PGx SNPs. A heatmap representation of the allele frequencies of predicted deleterious variants in pharmacogenes associated with different NIADs in Indians and other global populations. The associated genes are color coded based on the specific drug function. The number of drugs associated with each affected gene and for each drug class is indicated. GME, Greater Middle East; NIAD, non-insulin antidiabetic drug; PGx, pharmacogenetic; SNP, Single Nucleotide Polymorphism.

Genomic annotation of IndiGen variants identified 36 998 non-synonymous variants, of which 15 917 variants were predicted to be deleterious by at least two in silico functional prediction tools. An overlap of these variants with 94 NIAD-associated pharmacogenes yielded 796 potentially deleterious variants in Indians disrupting the function of 94 genes associated with 44 NIADs. Fifty-two of these variants are prevalent at over 1% effect AF among Indians (figure 3, online supplemental table S3). Of these, nine variants are known PGx variants listed in PharmGKB. This includes level 3 evidence PGx variants such as *SLC22A1* variants rs2282143 (IndiGen: 10%) and rs12208357 (IndiGen: 2.4%) associated with decreased metformin clearance and bioavailability, respectively, *CYP2C9* variant rs1799853 (IndiGen:3%) associated with increased efficacy/hypoglycemic risk with SU drugs and *SLCO1B1* variant rs4149056 (IndiGen: 5%) associated with improved repaglinide and rosiglitazone response.

10.1136/bmjdrc-2023-003769.supp3 Supplementary data

In addition, we report a predicted deleterious variant in *SLC47A2* gene, rs34399035, with 1.3% AF in Indians that could lead to altered metformin response. We also highlight five common likely deleterious SNPs in *CYP2D6* gene, rs1065852 (in *10, IndiGen: 19.3%), rs28371703 (in *74, IndiGen: 8.9%), rs1058172 (in *139 and *127, IndiGen: 7.8%), rs3915951 (IndiGen: 5%), rs1135828 (in *81 and *86 alleles, IndiGen: 2%) and rs77913725 (in *81 and *86 alleles, IndiGen: 2%), which could potentially affect the metabolism of drugs such as alogliptin, nateglinide, dapagliflozin and rosiglitazone.

In addition, we also identified two potentially deleterious variants, rs11568367 in *ABCB11* gene and rs1143671 in *SLC15A2* gene, which are prevalent at a frequency of 11% and 34%, respectively, in Indians. Loss or diminished function of *ABCB11* might impair the distribution of multiple drugs such as repaglinide, glimepiride, glyburide, glipizide, troglitazone and rosiglitazone while *SLC15A2* inhibition may affect transport of nateglinide, chlorpropamide, glyburide and tolbutamide. Similarly, our analysis revealed two putative deleterious polymorphisms, rs11568658 and rs11568694 in *ABCC4* gene, which could potentially affect nateglinide response. These variants show AFs of 8% and 3%, respectively, among Indians versus 5% and 0.7%, respectively, in global populations. We also report a predicted deleterious variant rs60140950 in *SLCO1B3* gene with 5% AF in Indians (1KGP3-ALL:0.07; gnomAD-ALL:0.11). *SLCO1B3* acts as a transporter for empagliflozin and pioglitazone. Some of these variants have already been implicated in the PGx of other drugs.

### NIAD pathways frequently disrupted in Indians

A Sankey pathway of 32 drugs associated with 30 genes that are potentially functionally hampered in at least 1% of Indians was generated (online supplemental figure S1). We identified 12 NIADs (carbutamide, glibornuride, glimepiride, glipizide, gliquidone, glisoxepide, metahexamide, tolazamide, repaglinide, rosiglitazone, pioglitazone and troglitazone), for which at least 1% of Indians show complete disruption of either of its target, enzyme or transport function. Interestingly, the sole metabolizer (*CYP2C9*, rs1799853) as well as transporter (*ABCB11*, rs11568367) genes for glimepiride are potentially hampered in 3% and 11% of Indians, respectively. Similarly, both transporters of troglitazone, rosiglitazone and repaglinide, *SLCO1B1* (rs4149056, rs201722521) and *ABCB11* (rs11568367) are potentially impaired with an AF of 5%, 3% and 11%, respectively, among Indians. Pioglitazone also showed both its transporters *SLCO1B3* (rs60140950) and *SLCO1B1* (rs4149056, rs201722521) potentially affected in 5.3%, 5% and 3% (AF), respectively, of Indians. An additional 10 drugs are also potentially affected by at least 50% disruption of any of its three functions at >1% frequency in Indians (online supplemental table S4). Three out of four targets of ⍺-glucosidase inhibitor miglitol (*GAA*, *GANAB* and *GANC*) were also frequently impaired among Indians at a frequency of 1.4%–6%.

10.1136/bmjdrc-2023-003769.supp4 Supplementary data

### Drug–drug interactions in diabetes therapy

**Figure 4:**
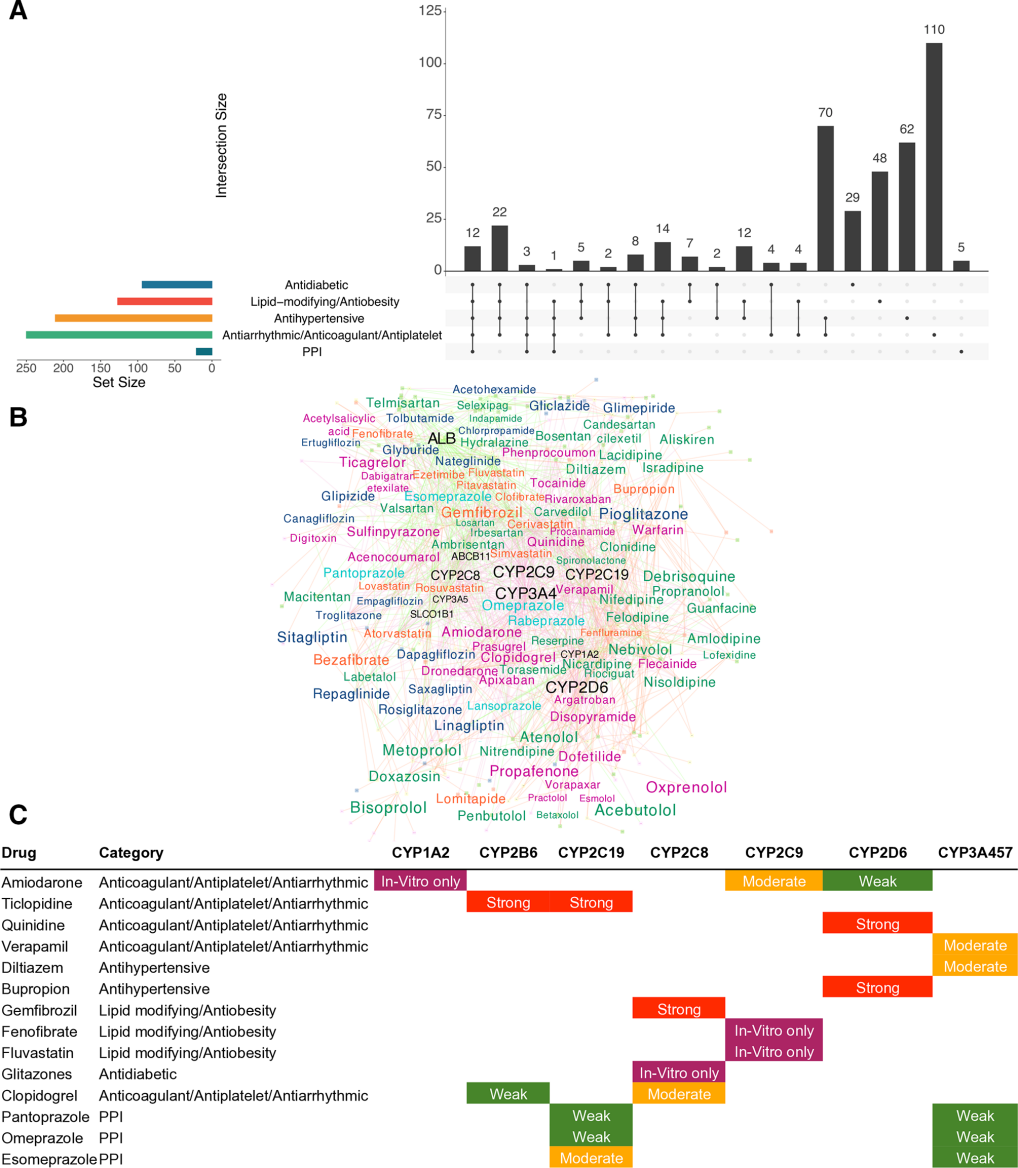
Genetic basis of DDIs in T2D therapy. (A) An UpSet plot depicting shared PGx genes associated with T2D therapy and treatment of metabolic disorders. (B) A network analysis of drug–gene interactions involved during polypharmacy in T2D therapy. The gene labels are highlighted in black and the drug labels are colored according to the disease category. The gene label sizes are proportional to the degree of the node (number of drug connections). The drug labels are sized according to the proportion of shared PGx genes associated with each drug (see Methods for details). (C) A table of validated CYP inhibitory actions of NIADs and associated polypharmacy drugs as per the Flockhart table of drug–gene interactions. A strong inhibitor is one that causes a>5 fold increase in the plasma AUC values or more than 80% decrease in clearance. A Moderate inhibitor is one that causes a>2 fold increase in the plasma AUC values or 50–80% decrease in clearance. A weak inhibitor is one that causes a>1.25 fold but<2 fold increase in the plasma AUC values or 20–50% decrease in clearance. AUC, area under the curve; DDI, drug–drug interaction; NIAD, non-insulin antidiabetic drug; PGx, pharmacogenetic; PPI, proton-pump inhibitor; T2D, Type 2 Diabetes.

Towards identifying pharmacogenetic (PGx) factors driving DDIs involving polypharmacy in diabetes patients, 242 metabolic disease drugs including lipid modifying/anti-obesity agents, antihypertensives, antiarrhythmic agents, antiplatelets, anticoagulants and proton pump inhibitors (PPIs) associated with 391 PGx genes were identified (online supplemental table S5). An overlap analysis with NIAD-associated genes revealed 12 genes, including seven enzymes and five transporters, which are shared by all drug categories (figure 4A). PPI-associated genes showed the maximum overlap (71%) with NIAD-associated genes followed by lipid-modifying/antiobesity drugs (38%), antihypertensives (25%) and antiarrhythmic/antiplatelet/anticoagulant agents (20%). This is highly relevant as the most coprescribed medications in Indian diabetes patients are hypolipidemics (72%), antihypertensives (68%), drugs for peptic ulcer (34.7%) and antiplatelets (10.7%).^35^

10.1136/bmjdrc-2023-003769.supp5 Supplementary data

A network analysis of shared genes highlighted *CYP3A4* followed by *CYP2C9*, *CYP2D6* and *CYP2C19* to be the most shared enzymes while *ABCB11* and *SLCO1B1* were the most shared transporters by different NIAD classes (figure 4B, online supplemental table S6). Based on the proportion of shared PGx genes, drugs such as pioglitazone, sitagliptin, repaglinide, linagliptin, rosiglitazone, gliclazide and glimepiride have the highest risk for DDIs with metabolic disease drugs.

10.1136/bmjdrc-2023-003769.supp6 Supplementary data

Our polypharmacy drug list also overlapped with several weak, moderate and strong inhibitors of CYP enzymes listed in the Flockhart table of experimentally validated CYP–drug interactions (figure 4C). For example, moderate inhibition of CYP2C9 activity by antiarrhythmic drug amiodarone indicates potentially increased risk of hypoglycaemia when coadministered with SUs similar to other CYP2C9 inhibitors.^36^ Similarly, use of saxagliptin and linagliptin (metabolized by CYP3A4, CYP3A5) along with CYP3A4/5 inhibitors such as antiarrhythmic agent verapamil or antihypertensive drug diltiazem could lead to increased plasma levels and higher risk for adverse events. CYP2C8 inhibitors such as lipid-modifying drug gemfibrozil (strong) and antiplatelet drug clopidogrel (moderate) could induce adverse events when consumed with repaglinide and TZDs, thereby requiring alternative drug/dose adjustment.

Genetic polymorphisms also influence the severity of DDIs.^14 29^ A focused literature search allowed us to identify polypharmacy drugs in our list that could potentially induce DDGIs in diabetes patients classified under three categories.^37^ Category 1 denotes DDGIs that magnify DDIs on the same pathway, while category 2 refers to those that magnify DDIs on different pathways and category 3 refers to those where the associated DDIs and genetic variants cause opposing effects. Known DDGIs include multiple SLC22A1-mediated interactions with metformin and SLCO1B1-mediated interactions with SUs and repaglinide (Online supplemental table S7). A total of 12 category 1, 6 category 2 and 1 category 3 DDGIs were predicted for various polypharmacy drugs and the NIADs. This includes prediction of an elevated risk for increased drug exposure during coadministration of PPIs (omeprazole, lansoprazole, rabeprazole and pantoprazole) and cilostazol with DPP-4I drugs, saxagliptin and linagliptin, which are exclusively metabolized by CYP3A4/3A5 as well as SU drug glyburide, for which CYP3A4 acts as the major metabolizer. These findings warrant further experimental validation in Indians given its substantial prevalence of *CYP2C19* variants (IndiGen-AF-*2:36%).

## Discussion

Despite a high proportion of type 2 diabetes patients with poor glycemic control in India, there is a dearth of PGx investigations in the Indian population to study the differential prevalence of known PGx variants. In the current study, we analyzed 1029 Indian whole genomes, one of the largest genomic repositories, to compile and assess the distribution of 76 known and 52 common predicted deleterious NIAD-associated PGx variants in Indians. We observed remarkable population-wise disparities in majority of the variant frequencies, in concordance with earlier multipopulation studies^38 39^ and also in the cumulative variant burden grouped by the minor allele’s impact on drug response. This finding amply highlights the importance of increasing population diversity in PGx research. Although most NIAD-associated PGx variants listed in PharmGKB have low level of evidence, Mannino *et al,* recently highlighted the most robust supporting evidence for variants in *OCTs*, *ATM* and *SLC2A2* loci with metformin response, *CYP2C9, TCF7L2, ABCC8, KCNJ11* and *IRS1* loci with SU response, *PPARG* locus with TZD response and *GLP1R* locus with DPP-4 inhibitors/GLP-1 analogues response.^9^

Metformin is the recommended first-line NIAD for type 2 diabetes in most guidelines across the world, including India. The organic cation transporters 1 and 2 (encoded by *SLC22A1* and *SLC22A2* genes) play vital roles in metformin’s renal transport, liver uptake and intestinal absorption and are, therefore, known modulators of metformin response. We reported an AF of 25% for a decreased metformin response-associated *SLC22A1* variant rs622342 (C) among Indians. An earlier Indian study has reported that carriers of AA genotype of the variant show 5.6 times better chance of response to metformin when compared with patients with CC genotype.^12^ However, this finding was not replicated in another South Indian study where two novel associations were reported with *SLC22A2* rs316019 and *SLC47A2* rs12943590.^13^ The authors also noted that the combined genotypes showed maximum average change in hemoglobin A1C (HbA1c) level. Overall, 18 out of 22 known metformin-associated variants were found among Indians with a median of 10 effect alleles in each individual. Given the contrasting impact of different variants, the individual response phenotype can only be effectively assessed by evaluating the net cumulative effect of the variant burden using population-specific or multiancestry effect sizes.^40^ Nevertheless, a simple measure of imbalance of total negative and positive allele counts per individual highlighted remarkable differences across populations, especially in case of metformin. Our analysis suggests better metformin response in Africans compared with other populations in concordance with previous reports.^41^ Our findings also suggest a lower metformin response in Indians and South Asians compared with other populations; however, this remains to be validated.

Sulfonylureas are the most widely prescribed second-line therapy in combination with metformin in India, also displaying substantial interindividual variation in its hypoglycaemic response among patients. The GG genotype of *TCF7L2* variant rs12255372, which was shown to favorably influence sulfonylurea response with its probable role in β-cell function among Indian patients, was found at 62% AF in Indians compared with 95% in East Asians.^11^ However, the same study failed to validate any association between *TCF7L2* variant rs7903146 and SU response among Indians. A recent meta-analysis has highlighted the association between decreased function *CYP2C9* alleles and increased risk of severe hypoglycaemia in patients treated with sulfonylureas.^42^ We observed a significant^43^ enrichment of CYP2C9 poor metabolizers in Indians compared with other populations (2%). A recent meta-GWAS on glycemic response to sulfonylureas highlighted a better response-associated variant, rs10770791 (C) in *SLCO1B1* gene, involved in regulation of SU transport that is marginally less prevalent in Indians compared with others (IndiGen:0.44, gnomAD-ALL:0.55).^43^ This variant was also linked to a DDGI with coprescription of statins.

Incretin-based therapies are known to demonstrate great efficacy in Indians through their ameliorative effect on β-cell function and are rapidly catching up with SUs as the preferred second-line treatment among Indians.^44^ The high prevalence (12%) of *GLP1R* variant rs692376112 (A), which is associated with decreased response to sitagliptin and vildagliptin, is, therefore, noteworthy and warrants clinical validation in Indians. Our Flockhart analysis also predicted increased drug exposure during coadministration of saxagliptin/linagliptin with moderate CYP3A4/5 inhibitors such as antiarrhythmic agent verapamil or antihypertensive drug diltiazem. Patel *et al* have confirmed the same where diltiazem use increased the area under the curve of saxagliptin by 109%.^45^ However, no dose-adjustment has been recommended for the use of saxagliptin with diltiazem or verapamil. Linagliptin, however, is only partially metabolized (19%) by CYP3A4 enzyme, thereby minimizing any impact of concomitant CYP3A4 inhibitors on its plasma levels.^46^ We also predicted a modest DDGI impact of antiplatelet drug cilostazol and PPIs on DPP-4Is in CYP2C19 PMs who constitute over 14% of Indians.

Although characterized by heterogeneous treatment response, novel NIAD classes such as SGLT2 inhibitors have no clinical annotations in PharmGKB, potentially due to insufficient reproducible evidence. Repaglinide, however, has several reported PGx variants, mostly identified from studies involving the Chinese population. Indians showed the lowest prevalence (Indigen:4%) of *KCNQ1* rs2237892 (T) associated with better repaglinide response by regulating insulin resistance compared with 35.5% in East Asians. Repaglinide’s increased susceptibility to DDIs owing to multiple shared PGx genes was also noted. Angiotensin II receptor blocker, irbesartan comedication reportedly increased repaglinide exposure exclusively in subjects with *SLCO1B1* c.521 TT genotype in Chinese population.^47^ Moreover, our drug pathway analysis predicted impaired transport of repaglinide and TZDs such as troglitazone, rosiglitazone and pioglitazone in at least 3% of Indians due to potentially deleterious variants in their transporter genes. Recent studies report that pioglitazone accounts for up to 10% of total diabetes prescriptions at tertiary care facilities in India while meglitinides share a negligible proportion.^44 48^

Polygenic risk scores have emerged as a promising tool to predict treatment outcomes across diseases. Very few studies have been reported for antidiabetic treatment and involve fewer variants.^49 50^ Josephine *et al* observed that patients in the top decile of type 2 diabetes polygenic score showed greater HbA1c reduction to sulfonylureas compared with those in the bottom decile. Although this approach holds great potential, knowledge of population-specific variant effect sizes is central to this approach. This further emphasizes the need for PGx validation studies in Indians.

The current study is one of the most comprehensive PGx surveys of known and predicted NIAD-associated PGx variants along with DDIs and DDGIs in the Indian population. We show that Indians and South Asians have a distinct NIAD PGx profile characterized by an extensive and excessive set of poor response alleles associated with various NIAD classes. Although categorized as low evidence or unvalidated, we cannot overlook the likelihood of a population-specific reduced drug response in atleast some NIADs. Our findings, therefore, provide a useful resource for driving future PGx studies in Indians while emphasizing the need to formulate country-specific policies and dosing guidelines for NIADs towards ensuring maximum efficacy and safety in Indians. The main limitation of our study is the lack of population-specific effect sizes for PGx variants, limiting the application of robust polygenic risk scores for drug response prediction. However, allele count differences between negative and positive response associated alleles revealed NIAD classes with a likely reduced response in Indians.

## Conclusion

Precision medicine for type 2 diabetes therapy remains a big challenge as opposed to its successes in monogenic diabetes owing to disease heterogeneity and complex pathophysiology, in addition to the racial/ethnic differences. Our findings highlight a comprehensive set of population-specific genetic factors that need to be scrutinized and leveraged to improve therapeutic response to diabetes therapy in Indians. The subsequent essential phase for clinical adoption involves validating the effect sizes of pertinent PGx variants within the Indian population and application of polygenic score-based approaches for better prediction of drug response traits and patient stratification.

## Data Availability

All data relevant to the study are included in the article or uploaded as supplementary information.
