# Supplementary material for: Landscape of pharmacogenetic variants associated with non-insulin antidiabetic drugs in the Indian population
Source: BMJ Open Diabetes Res Care. 2024 Mar 12;12(2):e003769. doi: 10.1136/bmjdrc-2023-003769 (PMC10936492; doi:10.1136/bmjdrc-2023-003769)
Supplement: Supplementary data [file bmjdrc-2023-003769supp001.pdf]

Supplemental Information

The Landscape of Pharmacogenetic Variants associated with Non-Insulin Antidiabetic drugs in the Indian population

Ambily Sivadas*[1], S Sahana[2,3], Bani Jolly[2,3], Rahul C Bhoyar[2], Abhinav Jain[2,3], Disha Sharma[2], Mohamed Imran[2,3], Vigneshwar Senthivel[2,3], Mohit Kumar Divakar[2,3], Anushree Mishra[2], Arpita Mukhopadhyay[1], Greg Gibson[4], KM Venkat Narayan[5], Sridhar Sivasubbu[2,3], Vinod Scaria[2,3] , Anura V Kurpad*[1]

[1]Division of Nutrition, St John's Research Institute, Bangalore, India

[2]CSIR Institute of Genomics and Integrative Biology, New Delhi, India

[3]Academy of Scientific and Innovative Research (AcSIR), Ghaziabad, Uttar Pradesh, India

[4]School of Biological Sciences, Georgia Institute of Technology, Atlanta, Georgia, USA

[5]Hubert Department of Global Health, Rollins School of Public Health, Emory University, Atlanta, Georgia, USA

[6]Department of Epidemiology, Rollins School of Public Health, Emory University, Atlanta, Georgia, USA

*Co-corresponding authors:

Anura Kurpad, MD, PhD, FRCP

Division of Nutrition, St John's Research Institute, Sarjapur Road, Bangalore, India. PIN: 560034. Phone: +91-80-49467000, Fax: +91-80-25501088

Email address: a.kurpad@sjri.res.in

Ambily Sivadas, PhD

Division of Nutrition, St John's Research Institute, Sarjapur Road, Bangalore, India. PIN: 560034. Phone: +91-80-49467000, Fax: +91-80-25501088

Email address: ambily.s@sjri.res.in

Keywords: Pharmacogenomics, Indians, South Asians, Diabetes, Non-insulin antidiabetic drugs, Population genomics, Drug-drug interactions, Drug-drug-drug-gene interactions

Running title: Pharmacogenomic landscape of T2D therapy in Indians

Supplemental Methods

Quality control

Genotype and individual level missingness tests (95%) and Hardy-Weinberg disequilibrium test ($p < 5 \times 10^{-7}$) were performed using PLINK v1.09(1) to obtain a final set of 53,672,515 variations.

Prediction of potential deleterious variants

The exonic variants were assessed for their functional impact using SIFT(2), PolyPhen2(3) and MutationTaster(4). Nonsynonymous exonic variants that were predicted deleterious (SIFT: Damaging; PolyPhen2: Probably Damaging and MutationTaster2: Disease_causing) by at least two tools were utilized for downstream analysis.

Annotation of known PGx variants

Clinical annotations were downloaded from PharmGKB (dated Feb 5, 2021) which included 4086 annotations associated with SNVs and 471 linked to haplotypes. The clinical annotations were overlapped with 44 anti-diabetic drugs to obtain 84 NIAD-associated clinical annotations, of which 9 were excluded for reasons including ultra-rare allele frequencies, explicit stating of a lack of impact on the glucose lowering effect of the drug, retirement on the annotation in the latest PharmaGKB version etc. For SNVs and indels, AFs for the Indian population along with other global populations were fetched directly from the IndiGen, 1000 Genomes Phase 3 (1KGP3) project, gnomAD (v2.1.1) and

Greater Middle East (GME) variome databases. For haplotypes, star alleles were called for each individual using Stargazer(5) and Cyrius(6) (for CYP2D6 alleles) on whole genome sequences from the IndiGen and 1KGP3 projects. Subsequently, their AFs were also estimated. Indian AFs were compared with the global allele frequencies (1KGP3-ALL and gnomAD-ALL) using fisher's exact test with false discovery rate (fdr) correction and visualized using R platform.

Sankey analysis

Flourish studio tool was used to visualize the drug function disruption pathway as a sankey diagram. Complete disruption of function refers to a predicted loss of function of all the genes associated with a particular function of a drug. For eg., if drug A has 4 associated transporters and all of them are disrupted commonly in the population (at a frequency of over 1 percent), we call that *complete* disruption of transport function for that gene. Similarly, 50% disruption refers to a predicted loss of function of at least 50% of the genes associated with a particular function of a drug.

Calculation of drug interaction score

The drug interaction score estimates the proportion of shared PGx genes associated with each drug. The score was computed as the cumulative average sum of the gene degrees associated with each drug.

$$\text{Drug interaction score} = \Sigma\, D_{enzyme\text{-}i} / N_{enzymes} + \Sigma\, D_{Target\text{-}i} / N_{Targets} + \Sigma\, D_{Transporter\text{-}i} / N_{Trasnporters}$$

where $D_{enzyme\text{-}i}$, $D_{Target\text{-}i}$ and $D_{Transporter\text{-}i}$ are the degrees of enzyme i, target i and transporter i associated with the drug and $N_{enzymes}$, $N_{Targets}$ and $N_{Trasnporters}$ are the total number of enzymes, targets and transporters associated with each drug. The interaction scores were subsequently normalized to the maximum score obtained for each node type (drug/gene).

Supplemental Tables

Supplemental Table S1: The list of NIADs and the associated pharmacogenes.

DBID	Name	Category	Targets	Enzymes	Transporters	Carriers
DB00284	Acarbose	α-glucosidase inhibitors	MGAM, AMY2A, GAA, SI,			
DB00491	Miglitol	α-glucosidase inhibitors	MGAM, GAA, GANAB, GANC,	AMY2A,		
DB04878	Voglibose	α-glucosidase inhibitors	MGAM,			
DB00331	Metformin	Biguanides	PRKAB1, ETFDH, GPD1,		SLC22A1, SLC22A2, SLC22A3, SLC47A1, SLC29A4, SLC47A2,	
DB01261	Sitagliptin	DPP4 inhibitors	DPP4,	CYP3A4, CYP2C8,	ABCB1, SLC22A8,	
DB04876	Vildagliptin	DPP4 inhibitors	DPP4,			
DB06203	Alogliptin	DPP4 inhibitors	DPP4,	CYP3A4, CYP2D6,		
DB06335	Saxagliptin	DPP4 inhibitors	DPP4,	CYP3A4, CYP3A5,	ABCC1, SLCO4C1, SLC22A8,	
DB08882	Linagliptin	DPP4 inhibitors	DPP4,	CYP3A4,	ABCB1, SLC22A1, SLC22A2, SLC22A3,	

DB12412	Gemigliptin	DPP4 inhibitors				
DB12625	Evogliptin	DPP4 inhibitors				
DB01276	Exenatide	GLP-1 analogues	GLP1R,	DPP4,		ALB,
DB06655	Liraglutide	GLP-1 analogues	GLP1R,	DPP4, MME,		ALB,
DB09043	Albiglutide	GLP-1 analogues	GLP1R,			
DB09045	Dulaglutide	GLP-1 analogues	GLP1R,			
DB09265	Lixisenatide	GLP-1 analogues	GLP1R,			
DB13928	Semaglutide	GLP-1 analogues	GLP1R,	DPP4, MME, LPL, AMY1A,		ALB,
DB00731	Nateglinide	Glinides	ABCC8, PPARG,	CYP2C9, CYP3A4, CYP3A5, CYP3A7, PTGS1, UGT1A9, CYP2D6,	ABCC4, SLC16A1, SLC15A1, SLC15A2, SLC22A6,	ALB, ORM1,
DB00912	Repaglinide	Glinides	ABCC8, PPARG,	CYP3A4, CYP2C8,	SLCO1B1, ABCB11,	ALB,
DB01252	Mitiglinide	Glinides	ABCC8, PPARG,	UGT1A3, UGT2B7,		
DB01278	Pramlintide	Other	CALCR, RAMP1, RAMP2, RAMP3,			
DB06292	Dapagliflozin	SGLT2 inhibitors	SLC5A2,	CYP1A1, CYP1A2, CYP2A6, CYP2C9, CYP2D6, CYP3A4, UGT1A9, UGT2B4, UGT2B7,	ABCB1,	
DB08907	Canagliflozin	SGLT2 inhibitors	SLC5A2,	UGT1A9, UGT2B4, CYP3A4,	ABCB1, ABCC2, ABCG2,	ORM1,
DB09038	Empagliflozin	SGLT2 inhibitors	SLC5A2,	UGT2B7, UGT1A3, UGT1A8, UGT1A9,	ABCB1, ABCG2, SLC22A8, SLCO1B1, SLCO1B3,	
DB11698	Ipragliflozin	SGLT2 inhibitors				
DB11827	Ertugliflozin	SGLT2 inhibitors	SLC5A2,	UGT1A9, UGT2B7, UGT1A1, UGT1A4,	ABCB1, ABCG2,	ALB,
DB12713	Sotagliflozin	SGLT2 inhibitors				
DB01382	Glymidine	Other	KCNJ1, ABCC8,			
DB00222	Glimepiride	Sulfonylureas	KCNJ11, KCNJ1, ABCC8,	CYP2C9,	ABCB11,	
DB00414	Acetohexamide	Sulfonylureas	KCNJ1,	CBR1, CYP2C9,		ALB,
DB00672	Chlorpropamide	Sulfonylureas	ABCC8,	CYP2C9, CYP2C19, PTGS1,	SLC15A1, SLC15A2, SLC22A6,	
DB00839	Tolazamide	Sulfonylureas	ABCC8,	CYP2C9,		
DB01016	Glyburide	Sulfonylureas	ABCC8,	CYP3A4, CYP2C9,	ABCC3,	ALB,

			ABCC9, ABCB11, ABCA1, KCNJ11, CPT1A, CFTR, TRPM4,	CYP2C19, CYP3A7, CYP3A5,	ABCB11, ABCB1, ABCC1, SLC15A1, SLCO1A2, SLC15A2, SLC22A6, ABCC2, ABCG2, SLC22A7, SLCO2B1,	
DB01067	Glipizide	Sulfonylureas	ABCC8, PPARG,	CYP2C9, UGT1A1,	ABCB11,	ALB,
DB01120	Gliclazide	Sulfonylureas	ABCC8, VEGFA,	CYP2C9, CYP2C19,		ALB,
DB01124	Tolbutamide	Sulfonylureas	ABCC8, KCNJ1,	CYP2C9, CYP2C8, CYP2C19, CYP2C18,	SLC15A1, SLCO1A2, SLC22A6, SLC15A2, SLCO2B1,	ALB,
DB01251	Gliquidone	Sulfonylureas	ABCC8, KCNJ8,	CYP2C9,		
DB01289	Glisoxepide	Sulfonylureas	KCNJ8,	CYP2C9,		
DB08962	Glibornuride	Sulfonylureas		CYP2C9,		
DB13406	Carbutamide	Sulfonylureas		CYP2C9,		
DB13675	Metahexamide	Sulfonylureas		CYP2C9,		
DB00197	Troglitazone	Thiazolidinediones	PPARG, ACSL4, SERPINE1, SLC29A1, ESRRG, ESRRA, PPARD, PPARA, GSTP1,	CYP3A4, CYP2C8, UGT1A1, CYP2C19, CYP19A1, CYP1A1, CYP2B6, CYP2C9, CYP3A5, CYP3A7, UGT1A3, UGT1A4, UGT1A6, UGT1A7, UGT1A8, UGT1A9, UGT1A10, UGT2B7, UGT2B15,	ABCB11, SLCO1B1,	
DB00412	Rosiglitazone	Thiazolidinediones	PPARG, ACSL4, PPARA, PPARD, RXRA, RXRB, RXRG,	CYP2C8, CYP2C9, PTGS1, CYP1A2, CYP2A6, CYP2D6, CYP3A4,	SLCO1B1, ABCB11,	ALB
DB01132	Pioglitazone	Thiazolidinediones	PPARG, MAOB,	CYP2C8, CYP3A4, CYP1A1,	SLCO1B3, SLCO1B1,	ALB,

Supplemental Table S2: The list of known NIAD-associated PGx variants in the PharmGKB database found in the Indian population (Indigen database) along with population-wise allele frequencies from the 1000 Genomes project, gnomAD

database and GME database. (Excel file)

Supplemental Table S3: The list of 52 predicted deleterious variants that are found to be prevalent at over 1% effect allele frequency in the Indian population along with population-wise allele frequencies from the 1000 Genomes project, gnomAD database and GME database. (Excel file)

Supplemental Table S4: Drugs associated with genes that are frequently disrupted in the Indian population at allele frequency > 1% (marked in red). (Excel file)

Supplemental Table S5: The list of metabolic disease drugs used for drug drug interaction analysis. (Excel file)

Supplemental Table S6: a. The list of drug - gene pairs associated with polypharmacy considered for network analysis; b. Calculation of weighted drug score; c. Calculation of normalized drug and gene scores. (Excel file)

Supplemental Table S7. List of potential drug–drug-gene interactions during polypharmacy in T2D therapy. The first and second columns list perpetrator and victim drugs with previously reported DDGIs along with predicted DDGIs for antidiabetes drugs in the last column.

Perpetrator Drug	Reported victim drug	Relevant genotypes	Predicted victim NIAD
Category 1 (Single gene- double hit)			
CYP2C9-mediated			
Amiodarone, clopidogrel, pantoprazole, losartan, irbesartan, fluvastatin, simvastatin	Vitamin-k antagonists (Acenocoumarol, warfarin, etc)(7)	CYP2C9*2/*3	SUs
Telmisartan	Fluvastatin(7)	CYP2C9*1/*3	SUs
CYP2D6-mediated			
Dronedarone, Amiodarone, Propafenone	Metoprolol(7)	CYP2D6 PM and/or IM	Alogliptin
Propafenone	Lidocaine, mexiletine(7)	CYP2D6 PM and/or IM	Alogliptin
Amiodarone	Flecainide(7)	CYP2D6 PM and/or IM	Alogliptin
Quinidine	Brofaromine, dextromethorphan, encainide, methoxyphenamine, mexiletine, procainamide, propafenone, R-flecainide, venlafaxine(7)	CYP2D6 PM and/or IM	Alogliptin
CYP2C19-mediated			
PPIs	Clopidogrel, Warfarin(7)	CYP2C19*2/*3	SUs
Clopidogrel, Ticlopidine	Omeprazole(7)	CYP2C19*2/*3	SUs
ABCB1-mediated			

Diltiazem	Cyclosporine(8)	ABCB1- rs1045642(C>T)	Sitagliptin, Dapagliflozin, Ertugliflozin, Canagliflozin
SLC22A1-mediated			
PPIs, verapamil, diltiazem, doxazosin, spironolactone, clopidogrel, rosiglitazone, quinine, tramadol, codeine	Metformin(8)	SLC22A1 - R61C, C88R , G401S, M420del, and G465R	
SLCO1B1-mediated			
Statins	SUs(9)	SLCO1B1 - rs10770791(T>C)	Rosiglitazone, Troglitazone, Pioglitazone
Gemfibrosil	Repaglinide(8)	SLCO1B1 - rs4149056(T>C)	Rosiglitazone, Troglitazone, Pioglitazone
Pravastatin	Olmesartan(8)	SLCO1B1 - rs4149056(T>C)	Repaglinide, Rosiglitazone, Troglitazone, Pioglitazone
Category 2 (Different genes - double hit)			
CYP2C19-mediated			
Omeprazole, Lansoprazole, Rabeprazole (CYP3A4/5/7 and CYP2C19 inhibitor)	Tacrolimus(10)	CYP2C19 IM/PM	Linagliptin, Sitagliptin
Pantoprazole (CYP3A4/5 and CYP2C19 inhibitor)	Atorvastatin(10)	CYP2C19 PM	Linagliptin, Sitagliptin
Diltiazem (CYP3A4/5/7 inhibitor)	Diazepam(10)	CYP2C19 PM	Linagliptin, Sitagliptin
Cilostazol (CYP3A4/5 inhibitor)	Clopidogrel(10)	CYP2C19 IM/PM	Linagliptin, Sitagliptin
PPIs and calcium channel blockers (CYP3A4/5 inhibitor)	Clopidogrel(8)	CYP2C19 PM	Linagliptin, Sitagliptin
CYP2D6-mediated			
Propafenone (CYP1A2 competitive inhibitor)	Caffeine(10)	CYP2D6 PM	
SLCO1B1-mediated			

Amlodipine (CYP3A4/5 inhibitor)	Simvastatin(8)	SLCO1B1 - rs4149056(T>C)	Linagliptin, Sitagliptin
Category 3 (single gene, opposing effects)			
PPIs	Clopidogrel(8)	CYP2C19 *17 (UM)	SUs

Supplemental Figures

Supplemental Fig. S1. Drug pathway map of NIAD-associated pharmacogenes that are most frequently functionally disrupted in the Indian population. A sankey chart representation of the affected drug-gene pairs grouped by different gene functions. The columns are arranged as drug names, followed by associated pharmacogenes sorted by their functional category: targets, enzymes and transporter/carriers. NA - Not Affected.

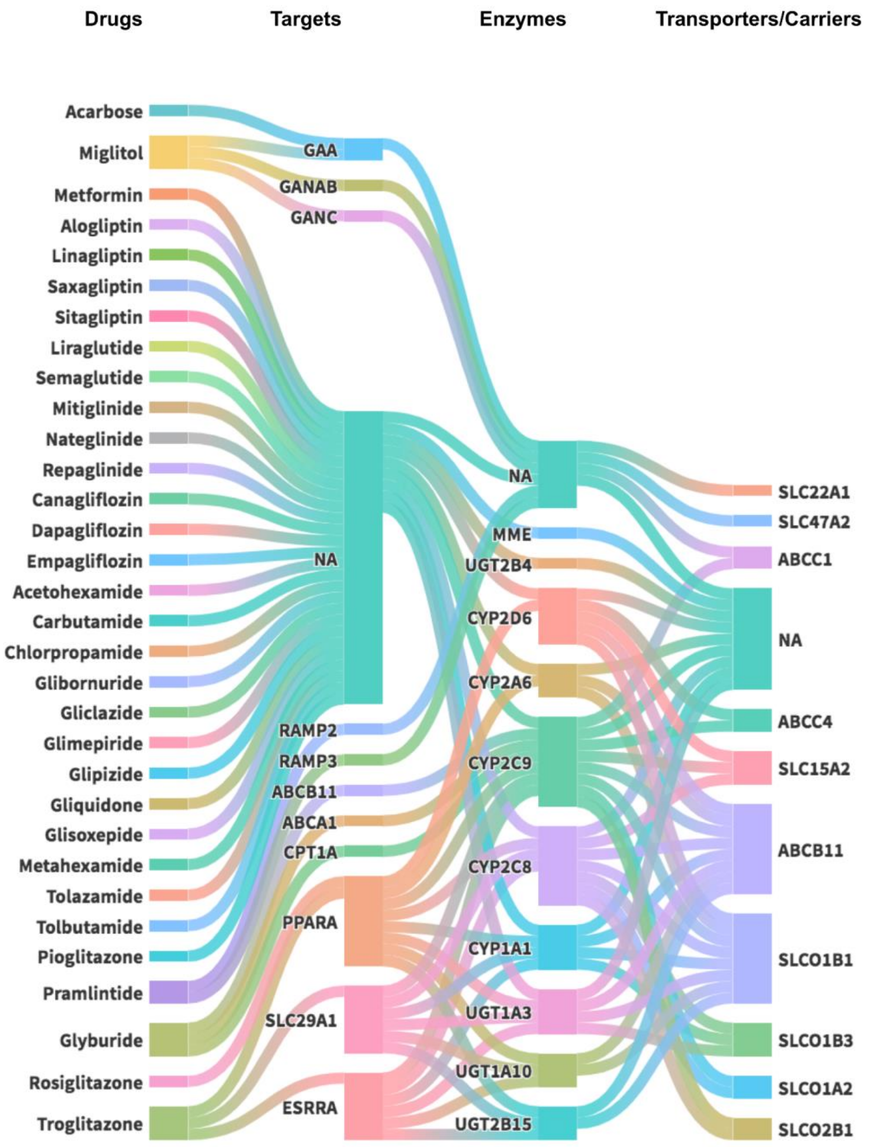

References

1. Purcell S, Neale B, Todd-Brown K, Thomas L, Ferreira MAR, Bender D, et al. PLINK: a tool set for whole-genome association and population-based linkage analyses. Am J Hum Genet. 2007 Sep;81(3):559–75.
2. Ng PC, Henikoff S. SIFT: Predicting amino acid changes that affect protein function. Nucleic Acids Res. 2003 Jul 1;31(13):3812–4.
3. Adzhubei I, Jordan DM, Sunyaev SR. Predicting functional effect of human missense mutations using PolyPhen-2. Curr Protoc Hum Genet. 2013;Chapter 7(SUPPL.76).
4. Schwarz JM, Cooper DN, Schuelke M, Seelow D. MutationTaster2: mutation prediction for the deep-sequencing age. Nat Methods. 2014 Apr 28;11(4):361–2.
5. Lee S been, Wheeler MM, Thummel KE, Nickerson DA. Calling Star Alleles With Stargazer in 28 Pharmacogenes With Whole Genome Sequences. Clin Pharmacol Ther. 2019 Dec 1;106(6):1328–37.
6. Chen X, Shen F, Gonzaludo N, Malhotra A, Rogert C, Taft RJ, et al. Cyrius: accurate CYP2D6 genotyping using whole-genome sequencing data. Pharmacogenomics J 2021 212. 2021 Jan 18;21(2):251–61.
7. Asiimwe IG, Pirmohamed M. Drug-Drug-Gene Interactions in Cardiovascular Medicine. Pharmgenomics Pers Med. 2022;15:879–911.
8. Malki MA, Pearson ER. Drug–drug–gene interactions and adverse drug reactions. Vol. 20, Pharmacogenomics Journal. Springer Nature; 2020. p. 355–66.
9. Dawed AY, Yee SW, Zhou K, Leeuwen N van, Zhang Y, Siddiqui MK, et al. Genome-Wide Meta-analysis Identifies Genetic Variants Associated With Glycemic Response to Sulfonylureas. Diabetes Care. 2021 Dec 1;44(12):2673.
10. Bahar MA, Setiawan D, Hak E, Wilffert B. Pharmacogenetics of drug-drug interaction and drug-drug-gene interaction: A systematic review on CYP2C9, CYP2C19 and CYP2D6. Vol. 18, Pharmacogenomics. Future Medicine Ltd.; 2017. p. 701–39.
